# COVID-19 and the HIV care continuum in Uganda: minimising collateral damage

**DOI:** 10.12688/aasopenres.13099.1

**Published:** 2020-07-02

**Authors:** Enock Kagimu, Emily M. Martyn, Jane Gakuru, John Kasibante, Morris K Rutakingirwa, Richard Kwizera, Kenneth Ssebambulidde, Darlisha Williams, Jayne Ellis, Fiona V Cresswell, David B. Meya

**Affiliations:** 1 Infectious Diseases Institute, College of Health Sciences, Makerere University, Kampala, Uganda; 2 North Middlesex University Hospital, London, UK, London, UK; 3 University of Minnesota, MN,USA, minneapolis, MINNESOTA, USA; 4 London School of Hygiene and Tropical Medicine, London, UK; 5 5MRC-UVRI-LSHTM Uganda Research Unit, Entebbe, Uganda

**Keywords:** COVID-19, HIV care, PLWHIV, Opportunistic infections, sub-Saharan Africa

## Abstract

The novel coronavirus, severe acute respiratory syndrome coronavirus 2 (SARS-CoV-2), has spread across the world within months of its first description in Wuhan, China in December 2019, resulting in an unprecedented global health emergency. Whilst Europe and North America are the current epicentres of infection, the global health community are preparing for the potential effects of this new disease on the African continent. Modelling studies predict that factors such as a youthful and rural population may be protective in mitigating the spread of coronavirus disease 2019 (COVID-19) in the World Health Organisation (WHO) African Region, however, with 220 million infections and 4.6 million hospitalisations predicted in the first year of the pandemic alone, fragile health systems could still be placed under significant strain. Furthermore, subsequent disruptions to the provision of services for people living with HIV, or at risk of acquiring HIV, are predicted to lead to an extra 500,000 adult HIV deaths and a 2-fold increase in mother to child transmission of HIV in sub-Saharan Africa in 2020-2021. Ignoring these predictions may have severe consequences and we risk “stepping back in time” in AIDS-related deaths to numbers seen over a decade ago. Reflecting on our current experience of the COVID-19 pandemic in Uganda, we explore the potential impact of public health measures implemented to mitigate spread of COVID-19 on the HIV care continuum, and suggest areas of focus for HIV services, policy makers and governments to urgently address in order to minimise the collateral damage.

## Introduction

Coronavirus disease 2019 (COVID-19), caused by the novel severe acute respiratory syndrome coronavirus 2 (SARS-CoV-2), was first described in Wuhan, China in December 2019 and to date there have been more than 9,912,223 confirmed cases, with at least 497,067 deaths, globally ^1^. Recent modelling studies predict fewer deaths and severe infections in Africa compared to the United States and Europe, potentially due to younger and more rural population. Despite this, it is predicted that within the first year nearly a quarter of Africa’s 1 billion population may be infected with the pandemic continuing for a longer period with 4.6 million hospitalisations, which will still pose a significant strain on health systems ^2, 3^. Sobering estimates from World Health Organisation (WHO) and UNAIDS suggest a disruption in antiretroviral therapy (ART) due to COVID-19 could lead to more than 500,000 extra deaths from AIDS-related opportunistic infections in sub-Saharan Africa and reverse gains made in preventing mother-to-child transmission of HIV, with an increase in new child infections of up to 104% in Uganda ^4^. These figures were described by Dr Tedros Adhanom Ghebreyesus, Director-General of the WHO as “stepping back into history” ^5^. In this commentary, we present Uganda as an example of a sub-Saharan African country who quickly enacted strict containment measures which have hopefully successfully curbed the spread of coronavirus, though may result in profound consequences on the HIV care continuum. We suggest globally applicable measures for HIV service providers, government and policy makers to urgently consider in order to minimise the lasting collateral damage to HIV prevention and treatment efforts threatened by this pandemic.

### COVID-19 challenges and the HIV Care Continuum in Uganda

Uganda has a population of almost 43 million people including 1.4 million people living with HIV (PLWHIV) ^6, 7^. Excellent progress has been made towards the UNAIDS “90-90-90” 2020 targets with 84% of the population aware of their HIV status and 87% of these on ART, of which 88% of people are virally suppressed. At its peak in the 1990’s, Uganda’s HIV prevalence was 18–30%, which was reduced to 5.7% in 2019, largely due to the roll out of antiretroviral therapy (ART) and extensive public health campaigns around the importance of HIV testing, initiating ART and counselling on drug adherence ^7^.

**Figure 1.**
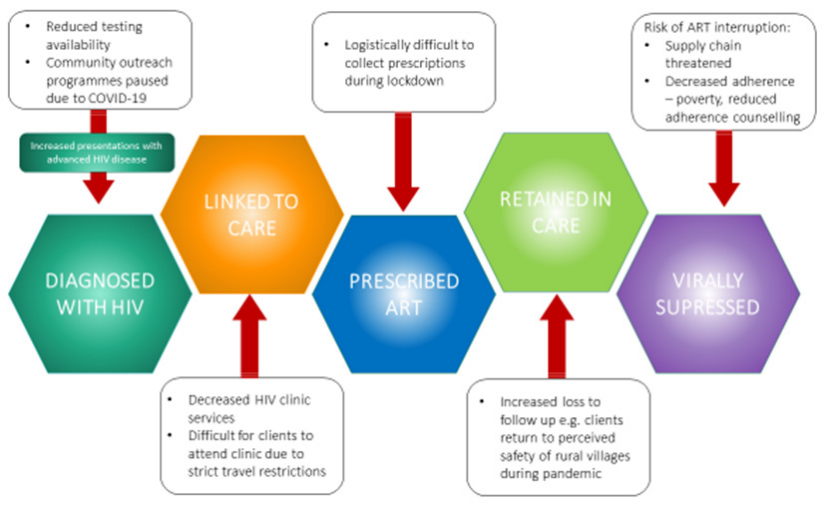
Challenges posed to the HIV care continuum by the COVID-19 pandemic. COVID-19 -Coronavirus disease (2019), ART- Antiretroviral therapy.

The current pandemic threatens to reverse these accomplishments. As soon as the first case of COVID-19 was identified in Uganda on 19 ^th^ March 2020, strict lockdown measures were enforced, including a ban on all public and private transport, night curfew, closure of schools, suspension of religious and social gatherings, and closure of non-essential shops and markets ^8^. Firstly, these measures present barriers to HIV testing, in particular initiatives for testing Uganda’s most vulnerable groups, including workplace testing, mobile mass testing campaigns and self-testing amongst fishermen, sex-workers and male partners of women attending antenatal care ^7^. For those with a new diagnosis of HIV, closures of clinics and the practicalities of leaving the house during lockdown prohibit essential linkage to care. However, our gravest concern is the real possibility of substantial ART interruption. In Uganda, this is likely multifactorial: supply chain issues as borders are closed, the inability of people to leave their homes to obtain essential medications, and people relocating to villages away from their HIV clinics. Additionally, there is the dire situation of poverty and hunger faced by many individuals, where mere survival rather than maintaining HIV care has become the priority. This disruption in ART access has very real consequences on those who were previously adherent to ART and virologically suppressed. Significant lapses in HIV virological suppression may result in increased community transmission of HIV in Uganda and other comparable settings, which would represent a catastrophic downstream effect of COVID-19 ( Figure 1).

### Minimising collateral damage: focus points for HIV service providers, policy makers and Government

**Figure 2.**
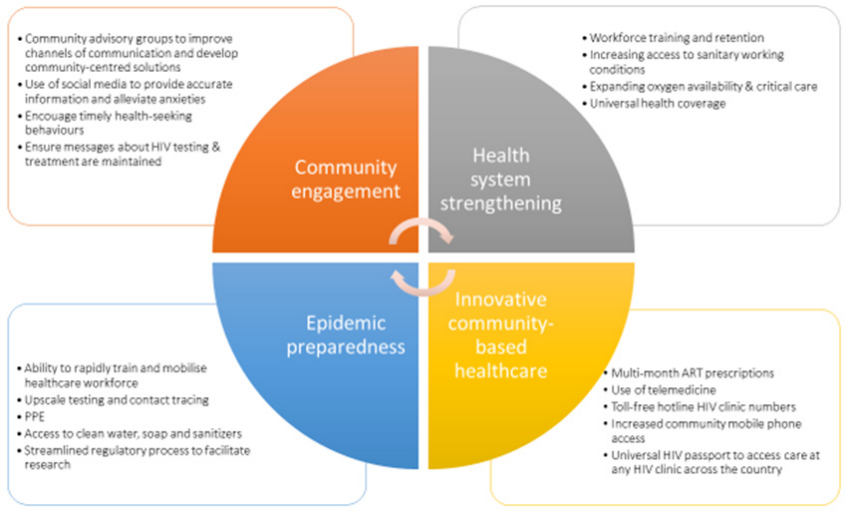
Suggested solutions to mitigate some of the major impacts of COVID-19 to HIV care and health systems and management of future pandemics. COVID-19 -Coronavirus disease (2019), ART- Antiretroviral therapy, PPE - Personal Protective equipment.

We suggest four focus points for HIV service providers, policy makers and governments to minimise collateral damage of the COVID-19 pandemic in Uganda, which are applicable to similar sub-Saharan African countries ( Figure 2).

***1. Increase in innovative, adaptive, decentralised, community-based care***. It is increasingly recognised that successful HIV care depends on adapting to the individual’s needs and expectations, especially in a pandemic ^9^. One such measure already recommended by WHO in response to COVID-19 is multi-month ART prescriptions for stable patients ^10^. However, ongoing maintenance of HIV care will require pro-active steps by HIV services, backed by adequate government funding and donor support. Adaptions to service delivery, such as telemedicine, must be incorporated so that clients can access essential care during prolonged lockdown. Hotlines should be set up in the event of an emergency, providing mobile credit allowance or toll-free numbers. Since less than 50% of the Ugandan population own a mobile phone, there should be provision of simple handsets or access to a shared community phone. For those who have relocated back to rural communities, issuing a universal HIV passport allowing clients to access medication at any clinic would prevent unintentional ART interruption. Ensuring PLWHIV have adequate food, water, soap and access to essential concomitant medications such as cotrimoxazole and fluconazole, are also essential and is something we have prioritised for the most vulnerable clients through donations at the Infectious Disease Institute, Kampala.

***2. Increased community engagement***. The community of PLWHIV, particularly in Uganda, has a strong history of activism and peer support. For example, many HIV services have community advisory boards comprised of patient representatives, religious and community leaders. In these challenging times, existing community networks, social media, radio and television should be leveraged to disseminate important public health messages pertaining to both COVID-19 prevention and HIV care, including HIV testing, ART adherence and encouraging people to access healthcare when needed, even during lockdown periods.

***3. Healthcare system strengthening***. Uganda has strong vertical health programmes such as HIV, TB and malaria control programmes; however, pandemics quickly expose lack of horizontal integration and a fragile surrounding healthcare infrastructure ^11^. COVID-19 will hopefully provide a wake-up call to government, donors and policy makers to invest in strengthening the Ugandan healthcare system so that essential HIV care can be maintained despite increased pressures posed by the pandemic. This should include increased training and retention of nursing, medical and auxiliary healthcare workers, ensuring adequate stock of medications and diagnostic laboratory equipment and provision of increased high-dependency unit beds and oxygen supply within government healthcare facilities, the only healthcare available to most Ugandans.

***4. Improved epidemic preparedness***. In the last 5 months, epidemic preparedness has become a top priority worldwide. In fact, at this time the global community might consider looking to Uganda’s experiences managing infectious epidemics such as HIV, Ebola, and Marburg for lessons in their own COVID-19 responses. Thankfully, COVID-19 spread in the African continent is predicted to be slower than in Europe and the United States, giving governments some time to prepare their responses ^2^. Nevertheless, ongoing efforts are required to upscale testing and contact tracing, rapidly train and mobilise a COVID-19 workforce, source adequate personal protective equipment (PPE) and ensure adequate access to adequate clean water, soap, and sanitizers for both healthcare facilities and the general population. In addition, emphasis should be placed on mobilisation of funds and streamlined regulatory processes to facilitate context-specific research on the epidemiology and clinical course of COVID-19 in PLWHIV, to avoid reliance on research findings being extrapolated from the global North.

## Conclusion

Failing to recognise the potential impact of the current pandemic on the HIV care continuum in Uganda, and similar sub-Saharan African countries, could result in a huge upsurge in HIV transmission and deaths in the months and years to come. In addition, increasing global travel, urbanization and changes in land use mean that future pandemics are almost inevitable ^12^. Substantial action must be taken now by HIV service providers, policy makers and governments in sub-Saharan Africa to create innovative, sustainable and effective solutions to prevent us “stepping back in time” in HIV care and minimise the collateral damage of COVID-19 on the HIV care continuum.

## Data availability

### Underlying data

No data are associated with this article.
