# Supplementary material for: COVID-19 and the HIV care continuum in Uganda: minimising collateral damage
Source: AAS Open Res. 2021 Mar 24;3:28. Originally published 2020 Jul 2. [Version 2] doi: 10.12688/aasopenres.13099.2 (PMC11208847; doi:10.12688/aasopenres.13099.2)
Supplement: Peer review history

# Reviewer report: Reviewer response for version 2

**DOI:** 10.21956/aasopenres.14316.r31422

**Published:** 2024-06-26

**Authors:** Warren Parker

**Affiliations:** 1 Centre for Communication Media and Society, University of KwaZulu-Natal, Durban, South Africa

Overall the open letter in its current form is fine. Obviously, it is specific to a point and time in the Ugandan COVID-19 epidemic, and the insights and recommendations are relevant to that time frame rather than being a contemporary or retrospective analysis. In any event, the points made are relevant and merit publication. It will be useful to include at the end of the letter, a paragraph stating the time period that these recommendations were made, indicating that they are valid to that period and subsequent unfolding of events are not taken into account. Also, in the article, there is a reference to "In the last 1 year" under point 4. This time period should be clarified.

Does the article adequately reference differing views and opinions?

Yes

Are all factual statements correct, and are statements and arguments made adequately supported by citations?

Yes

Is the Open Letter written in accessible language?

Yes

Where applicable, are recommendations and next steps explained clearly for others to follow?

Yes

Is the rationale for the Open Letter provided in sufficient detail?

Yes

Reviewer Expertise:

Public health, sociology of health, extensive experience in HIV pandemic response, and COVID-19 response.

I confirm that I have read this submission and believe that I have an appropriate level of expertise to confirm that it is of an acceptable scientific standard.

# Reviewer report: Reviewer response for version 2

**DOI:** 10.21956/aasopenres.14316.r28519

**Published:** 2021-03-26

**Authors:** Kristen D. Krause

**Affiliations:** 1 Center for Health, Identity, Behavior and Prevention Studies (CHIBPS), Rutgers School of Public Health, Newark, NJ, USA

I have no further comments to make at this time. Thank you for the opportunity to re-review.

Does the article adequately reference differing views and opinions?

Yes

Are all factual statements correct, and are statements and arguments made adequately supported by citations?

Partly

Is the Open Letter written in accessible language?

Yes

Where applicable, are recommendations and next steps explained clearly for others to follow?

Yes

Is the rationale for the Open Letter provided in sufficient detail?

Yes

Reviewer Expertise:

HIV/AIDS, aging, resilience, LGBTQ health disparities, psychosocial stressors, housing instability.

I confirm that I have read this submission and believe that I have an appropriate level of expertise to confirm that it is of an acceptable scientific standard.

# Reviewer report: Reviewer response for version 1

**DOI:** 10.21956/aasopenres.14198.r28176

**Published:** 2020-12-16

**Authors:** Kristen D. Krause

**Affiliations:** 1 Center for Health, Identity, Behavior and Prevention Studies (CHIBPS), Rutgers School of Public Health, Newark, NJ, USA

Thank you for the opportunity to review this commentary on minimizing the collateral damage of the HIV care continuum due to COVID-19. The authors present a clear and informative overview of the myriad of challenges (figure 1) and solutions to mitigate the impact (figure 2) of COVID-19. This is a noteworthy contribution to the literature given Uganda’s success in almost meeting the UNAIDS 90-90-90 targets. In addition to the comments provided by our colleague, Tolulope Tokunyori Oladele, I have a few minor suggestions that could strengthen this manuscript.

**Abstract:**

 Determine whether the third sentence should be re-worded to say: "Modelling studies predict that factors such as youthful and rural populations may be protective in mitigating the spread of COVID-19 in the World Health Organisation (WHO) African Region…." instead of "a youthful and rural population."

**Introduction/Main text:**

 Similar to the abstract, in the second sentence, I think there population should be plural OR it should read "a younger and more rural population".

 1. Increase in innovative, adaptive, decentralised, community based care.

 - Are any of these recommendations based on interventions that have been implemented in some capacity in Uganda or anywhere else? If so, it’d be helpful to cite or reference them so readers can easily find the information and resources (e.g. telemedicine in rural settings and HIV passport usage).

 This recommendation could be applied throughout other sections where applicable.

Does the article adequately reference differing views and opinions?

Yes

Are all factual statements correct, and are statements and arguments made adequately supported by citations?

Partly

Is the Open Letter written in accessible language?

Yes

Where applicable, are recommendations and next steps explained clearly for others to follow?

Yes

Is the rationale for the Open Letter provided in sufficient detail?

Yes

Reviewer Expertise:

HIV/AIDS, aging, resilience, LGBTQ health disparities, psychosocial stressors, housing instability.

I confirm that I have read this submission and believe that I have an appropriate level of expertise to confirm that it is of an acceptable scientific standard, however I have significant reservations, as outlined above.

# Response

**Published:** 2021-02-07

**Authors:** Enock Kagimu

**Affiliations:** Infectious diseases institute Makerere University, Uganda

Reviewer 2 (Kristen D. Krause)

 We sincerely thank the reviewers for their expert critique and constructive suggestions. We have responded to each comment in turn.

**Abstract:**

**Determine whether the third sentence should be re-worded to say: "Modelling studies predict that factors such as youthful and rural populations may be protective in mitigating the spread of COVID-19 in the World Health Organisation (WHO) African Region…." instead of "a youthful and rural population."**

 This has been adjusted.

**Introduction/Main text:**

**Similar to the abstract, in the second sentence, I think there population should be plural OR it should read "a younger and more rural population".**

 Article “a” has been added to the sentence

**1. Increase in innovative, adaptive, decentralised, community based care.**

**- Are any of these recommendations based on interventions that have been implemented in some capacity in Uganda or anywhere else? If so, it’d be helpful to cite or reference them so readers can easily find the information and resources (e.g. telemedicine in rural settings and HIV passport usage).**

**This recommendation could be applied throughout other sections where applicable.**

 Thank you for this comment. Yes, some of these recommendations have been tried out in Uganda though not much data captured for good referencing as some are still being tried out where as some are suggestions for better improvement.

# Reviewer report: Reviewer response for version 1

**DOI:** 10.21956/aasopenres.14198.r27657

**Published:** 2020-08-17

**Authors:** Tolulope Tokunyori Oladele

**Affiliations:** 1 Community Prevention and Care Services Department, National Agency for the Control of AIDS (NACA), Abuja, Nigeria

The article highlights the need for a continued focus on HIV despite the ongoing COVID-19. This is to prevent a reversal of gains already achieved in the fight against HIV citing Uganda as an example. A framework describing how this can happen is provided. Four focus point which HIV service providers, managers and government should note is provided. The article is well written, in clear English and easy to understand.

 Addressing the following points will improve the quality of the article:

**Introduction:**

1. The number of cases and deaths should be updated to the most recent figures. 
2. The article "a" should come before "younger" in Line "4".
3. Though the article makes reference to the fact that Uganda may have successfully curbed the spread of COVID-19, there is limited evidence such as " *delayed community transmission, limited number of cases, reducing new infections*" to support the statement. These should be included.

**COVID-19 challenges and the HIV Care Continuum in Uganda:**

1. The more general abbreviation for "people living with HIV is PLHIV", the authors may consider its usage.
2. The phrase " and instituted control measures" should be added" to the statement "the current pandemic threatens to reverse these accomplishments" as it is the lock-down measures such as restriction of movement that have  greatly impacted on access to HIV services.
3. The statement on substantial ART interruption should be strengthened by making reference to the possible global shortage of drugs such as" Tenofovir-Lamivudine-Dolutegravir and Lopinavir/ritonavir" - *https://bestlifeonline.com/coronavirus-medicine-shortages/,* https://www.state.gov/wp-content/uploads/2020/06/06.10.2020_PEPFAR-Technical-Guidance-During-COVID.pdf.
4. Secured funding is a critical component of minimizing collateral damage to HIV. The authors should consider bringing out this point separately from **focus point 1** - increase in innovative, adaptive, decentralized, community-based care.

Does the article adequately reference differing views and opinions?

Yes

Are all factual statements correct, and are statements and arguments made adequately supported by citations?

Partly

Is the Open Letter written in accessible language?

Yes

Where applicable, are recommendations and next steps explained clearly for others to follow?

Yes

Is the rationale for the Open Letter provided in sufficient detail?

Yes

Reviewer Expertise:

HIV/AIDS, Health Economics, Obstetrics & Gynaecology

I confirm that I have read this submission and believe that I have an appropriate level of expertise to confirm that it is of an acceptable scientific standard, however I have significant reservations, as outlined above.

# Response

**Published:** 2021-02-07

**Authors:** Enock Kagimu

**Affiliations:** Infectious diseases institute Makerere University, Uganda

Reviewer 1 (Tolulope Tokunyori Oladele):

 We sincerely thank the reviewers for their expert critique and constructive suggestions. We have responded to each comment in turn.

**Introduction:**

- **The number of cases and deaths should be updated to the most recent figures.**
  These have been updated.

 

1. **The article "a" should come before "younger" in Line "4".**

Article “a” has been added.

  

- **Though the article makes reference to the fact that Uganda may have successfully curbed the spread of COVID-19, there is limited evidence such as "** ***delayed community transmission, limited number of cases, reducing new infections*** **" to support the statement. These should be included.**

Thank you for the suggestion, this statement has been added.

**COVID-19 challenges and the HIV Care Continuum in Uganda:**

- **The more general abbreviation for "people living with HIV is PLHIV", the authors may consider its usage.**

This has been changed to PLHIV throughout the paper

  

- **The phrase " and instituted control measures" should be added" to the statement "the current pandemic threatens to reverse these accomplishments" as it is the lock-down measures such as restriction of movement that have greatly impacted on access to HIV services.**

This has been added to that line.

  

- **The statement on substantial ART interruption should be strengthened by making reference to the possible global shortage of drugs such as" Tenofovir-Lamivudine-Dolutegravir and Lopinavir/ritonavir" -** ***https://bestlifeonline.com/coronavirus-medicine-shortages/,******* **https://www.state.gov/wp-content/uploads/2020/06/06.10.2020_PEPFAR-Technical-** **Guidance-During-COVID.pdf** **.**

Thank you for the suggestion, this has been expounded and a reference to this added.

  

- **Secured funding is a critical component of minimizing collateral damage to HIV. The authors should consider bringing out this point separately from** **focus point 1** **- increase in innovative, adaptive, d** **ecentralized, community-based care.**

Thank you our dear reviewer, the point of secured funding has been noted in the epidemic preparedness section.
